# Adherence to the Healthy Nordic Food Index in the Norwegian Women and Cancer (NOWAC) cohort

**DOI:** 10.29219/fnr.v62.1339

**Published:** 2018-09-12

**Authors:** Torill M. Enget Jensen, Tonje Braaten, Bjarne Koster Jacobsen, Runa Borgund Barnung, Anja Olsen, Guri Skeie

**Affiliations:** 1 Department of Community Medicine, Faculty of Health Sciences, UiT-The Arctic University of Norway, Tromsø, Norway; 2 Danish Cancer Society Research Center, Institute of Cancer Epidemiology, Danish Cancer Society, Copenhagen 2100, Denmark

**Keywords:** healthy Nordic diet, dietary index, dietary pattern regional diet, the environmental impact of foods, energy adjustment

## Abstract

**Background:**

High adherence to the Healthy Nordic Food Index has been associated with better health outcomes, but the results have not been consistent. The association between high adherence and higher intake of energy and healthy and less healthy foods has been persistent across countries, highlighting the need to examine potential confounding by energy intake.

**Objective:**

This study aimed to examine energy-adjusted dietary factors and lifestyle factors related to the index in a Norwegian context.

**Design:**

The study was cross-sectional within the Norwegian Women and Cancer cohort and included 81,516 women aged 41–76. Information about habitual food intake was based on a food frequency questionnaire (FFQ). The index incorporated six food groups (fish, root vegetables, cabbages, apples/pears, whole grain bread, and breakfast cereals). Ordered trend and regression analyses were performed to assess the association between the index and lifestyle and dietary factors with energy-adjusted models.

**Results:**

Nearly one out of four women (22.8%) had low adherence, 49.0% had medium adherence, and 28.2% had high adherence to the index. Intake of energy and of both healthy and less healthy foods increased with increased adherence. Energy adjustment removed the associations between less healthy foods and high adherence and demonstrated a better dietary composition in high adherers. The healthy Nordic foods contributed more to the total food intake in high versus low adherers, and high adherence was associated with a healthier lifestyle.

**Conclusion:**

High adherence was associated with a healthier lifestyle, both concerning diet and other factors. Energy adjustment of potential confounding foods removed associations between high adherence and less healthy foods. The Nordic foods accounted for a larger fraction of the diet among high adherers, at the expense of other healthy foods. Careful adjustment for confounders is warranted when assessing associations between the index and health outcomes.

## Popular scientific summary

- This study assessed the dietary composition and lifestyle factors associated with adherence to the Healthy Nordic Food Index by energy-adjusted methods.
- Energy-adjustment pointed to a better dietary composition among high adherers.
- High adherers had a larger fraction of healthy Nordic foods at the expenses of other healthy foods in the diet.
- High adherers had an overall healthier lifestyle.
- Careful adjustment for confounders is warranted when assessing associations between the index and health outcomes.

The Mediterranean diet has been related to improved health since the first major studies of the food patterns typical of Crete in the 1960s (1). This dietary pattern consists primarily of plant foods (i.e. fruit, vegetables, whole grain, potatoes, beans, nuts, and seeds), moderate amounts of fish and poultry, low amount of red meat, and fat primarily from olives, and is strongly associated with reduced cardiovascular risk factors and disease (1–3). The use of indices that measure dietary patterns, such as in the studies on the Mediterranean diet, has become quite widespread in nutritional research (4). In recent years, there has been a growing interest in studying traditional Nordic foods by similar methods in order to investigate whether healthy regional based diets defined by an *a priori* index could display similar health benefits as the Mediterranean diet (5–8). In this context, several diet scores measuring adherence to healthy aspects of a Nordic diet have been developed, such as the Healthy Nordic Food Index, the New Nordic Diet, and the Baltic Sea Diet Score (7–9). High adherence to any of the three indices is associated with a more physically active lifestyle, and by design, high adherers have a higher intake of healthy foods such as whole grains, fish, fruits, and vegetables and thereby of essential nutrients. However, high adherence has been associated with a higher energy intake in all three indices, and with a higher intake of less healthy foods such as red meat and processed meat, and sweets in the Healthy Nordic Food Index and in the New Nordic Diet, and with a higher level of sodium in the Baltic Sea Diet Score. For individuals with higher energy requirements, and consequently a higher food intake, it could be easier to surpass the cutoff values and thereby get a high index score even with a less balanced diet. This is a general problem in studies on indices measuring dietary patterns, and this is why energy adjustment is recommended in these types of studies (10). The Healthy Nordic Food Index has not been investigated in a Norwegian context, but it is desirable to do so as high score on the index in some (11–14), but not all (15, 16), studies has been linked to lower risk of myocardial infarction, stroke, type-2 diabetes, and colorectal cancer in women in other countries. Furthermore, a new WHO report evaluated the health effects associated with a healthy Nordic diet and encourages the Nordic countries to investigate how it can be transformed into dietary advice that can be implemented in the population (17). In order to evaluate the effect of this, there is a need for baseline documentation and generally better understanding of factors related to the healthy Nordic diet in all Nordic countries.

The items included in the original Healthy Nordic Food Index (i.e. rye bread, fish, apples and pears, root vegetables, cabbages, and oatmeal) were chosen due to their positive association with health outcomes, the ability to be produced in the Nordic nature without the use of external energy, traditional use as foods in the region (e.g. not as spices), and availability in the FFQ used in the study (7). A diet based on local produce and food traditions is considered easier to comply with and takes the environmental impact of foods into account (14, 15, 18). This study aimed to describe how the Healthy Nordic Food Index was adapted to the information included in the Norwegian Women and Cancer (NOWAC) cohort and to describe the relationships between the adherence categories on the Healthy Nordic Food Index and the energy adjusted dietary composition and lifestyle factors in the NOWAC cohort.

## Materials and methods

### Participants

The NOWAC cohort is a prospective nationwide study with more than 170,000 participants (19). In short, the cohort recruitment took place from 1991 to 2007 in batches consisting of women randomly drawn from the central national population registry. Participants answered a self-administered questionnaire about hormonal and reproductive factors, smoking, alcohol, tanning habits, socio-economic conditions, height and weight, physical activity, participation in mammography screening, breast cancer in the family, other diseases, and self-reported health. Follow-up questionnaires were mailed to some of the participants. A majority of the questionnaires included four pages with food frequency questions. The baseline for this study is partly the first NOWAC mailing from 1996 to 1997 and 2003 to 2004 (response rate of 57 and 48%, respectively), and partly the second mailing (follow-up questionnaire) from 1998 to 99 to those enrolled in 1991 to 1992, who at enrolment had not answered an FFQ questionnaire (response rate of 81%). In total, this cohort comprises 101,321 women aged 41–76 at baseline, who answered questionnaires that included the food frequency questions. Participants with missing data on food items included in the Healthy Nordic Food Index (*n* = 3,913); with an extreme energy intake either <2,500 kJ (*n* = 924) or >15,000 kJ (*n* = 138) (20); or with missing data on height (*n* = 861), weight (*n* = 1,229), smoking status (*n* = 1,511), physical activity (*n* = 7,198), or years of education (*n* = 4,031) were excluded, leaving 81,516 participants for the analyses.

The NOWAC cohort has received approval for the collection and storing of questionnaire information. All data are stored and handled according to the permission given by the Norwegian Data Inspectorate. Participants have given informed consent, and ethical approval for the NOWAC cohort has been obtained from the Regional Committee for Medical and Health Research Ethics (REK).

### Dietary assessment

Diet was assessed using a semi-quantitative FFQ. The FFQ was designed to capture the typical diet during the past year, covering traditional foods in Norway with special emphasis on fish consumption (21). The response options were given in fixed frequencies and quantities check-boxes, with 4–7 frequency categories (e.g. carrots: never/seldom, 1–3 per month, 1 per week, 2 per week, 3 per week, 4–5 per week, and 6–7 per week). For some food items, an additional question concerning the typical amount consumed per occasion (portion size) was reported as natural units such as slices of bread, florets of broccoli and number of potatoes, or household units such as tablespoons, with alternatives ranging from 3 to 5 (e.g. carrots: 1/2 a carrot, 1 carrot, 1½ carrots, and 2+ carrots). The Norwegian Weight and Measurement Table, which has standardized portion sizes and weights, was used to convert the consumption of food items to grams (22). Information about energy and nutrient content in foods was obtained from the Norwegian Food Composition Database (23). The calculations of daily intake of food items, energy, and nutrients were done using a statistical program for SAS (SAS Institute Inc., Cary, NC, USA) developed at the Department of Community Medicine, University of Tromsø, for the NOWAC cohort. Missing values were substituted by conservative estimations, missing frequencies were treated as no consumption, and missing portion sizes were assumed to reflect the smallest portion size asked for. Food groups such as apples/pears were divided into single food items on the background of frequency weights obtained from a 24-h dietary recalls study within the NOWAC cohort (24).

### Absolute and relative nutrient intakes

Total energy intake were calculated in kilojoule (KJ). The contribution of macronutrients (protein; carbohydrates; total fat; polyunsaturated-, monounsaturated-, saturated-, and trans-fatty acids; and alcohol) was calculated as energy percentages (E%) of total energy intake and compared across adherence categories. The energy-adjusted intake of food items/nutrients was calculated by absolute intake of the food items/nutrients divided by energy intake (KJ) and scaled to intake per 7 MJ, which was the median energy intake in the cohort. This energy intake was chosen to compare absolute and energy-adjusted intake on the same relative scale.

### The Healthy Nordic Food Index

The Healthy Nordic Food Index, first developed by Olsen et al., was applied as closely as possible for comparability with previous studies using the index (7). Six food groups were included in the index: fish, root vegetables (carrots and swede), cabbages (cabbage, broccoli/ cauliflower), apples/pears, whole grain bread, and breakfast cereals. Due to the available questions in the FFQ used in the NOWAC cohort, and to some extent differences in food culture between Denmark and Norway, the original rye bread category was replaced by whole grain bread, and breakfast cereals (breakfast cereals/oatmeal/muesli) replaced the original oatmeal category (Table 1). The index components fish, root vegetables, and cabbages were based on several questions in the FFQ, whereas information on intake of whole grain bread, breakfast cereals, and apples/pears originated from single question. Table 1 shows the food items in the FFQ that were included in the six food groups incorporated in the index. To compute the index score for each participant, the intake of each food item included in the index was divided by the cohort median to assign each participant either 1 point if they were equal to or above the study median, or 0 point if below the study median. For breakfast cereals, the median consumption was 0, so 1 point was given to the participants who consumed any breakfast cereals. Finally, the assigned points for the six food groups were summed up, giving each participant a score between 0 and 6.

**Table 1:** Food items from the food frequency questionnaire included in the calculation of the Healthy Nordic Food Index in the Norwegian Women and Cancer cohort

Index food category (number of questions)	Description of food items included in the index food category	Changes	Scoring criteria	Separate portion size question
Fish (12)			Median	
Fish as a main course (6)	Poached cod, pollock, haddock, Pollack Fried cod, pollock, haddock, Pollack Catfish/flounder/redfish Salmon/trout Mackerel Herring		Subcohorts 4 and 5 include a category for ‘other fish’	Yes
Fish spread (6)	Mackerel in tomato/smoked Mackerel Caviar Herring/anchovies Salmon, smoked/cured Other fish spread	Subcohort 1 includes questions on tuna and sardine. Subcohort 1 and 2 include three categories, that is, mackerel in tomato/smoked mackerel, caviar, and other fish spread.		No*
Root vegetables (2)	Carrots Swede		Median	Yes
Cabbage (2)	Cabbage Broccoli/cauliflower		Median	Yes
Apples/pears (1)	Apples/pears		Median	No
Whole grain bread (1)	Whole grain bread	Subcohorts 4 and 5 include a question on kneipp bread (partly whole grain).	Median	No
Breakfast cereals (1)	Cereal/oatmeal/muesli		Consumers/non-consumers	No

* Correspond to the number of slices of bread with fish spread in the FFQ.

Participants with 0–1 points were defined as low adherers, those scoring 2–3 points were defined as medium adherers, and those scoring 4–6 points were defined as high adherers (7).

### Foods and nutrients not included in the index

Comparison of the absolute intake (gram/day) and the energy-adjusted intake (gram/7 MJ) of some food items outside the index that contribute to the total energy intake was included in the analysis to get a better understanding of the dietary composition associated with adherence to the index. Some of these food items are not associated with a clear positive or negative health effect (i.e. milk and milk products, chicken, and potatoes), whereas red meat and processed meat, sodium and added sugar are considered less healthy, and other fruits (orange, banana, and ‘other fruits’) and other vegetables (tomato, salad, and two general categories ‘other vegetables’ and ‘vegetable mix’) are considered healthy, but not incorporated in the index.

Fiber (gram) and sodium (milligram) were calculated as absolute intake (gram or milligram/day) and as energy-adjusted intake (gram or milligram/7 MJ). Intake of some essential micronutrients (vitamin D, folate, selenium, zinc, and iron) was included on the basis of surveys in the Nordic countries which have shown that the recommended intake of these nutrients could be difficult to fulfil through the diet alone (25). Micronutrients were calculated as absolute intake (unit/day), and compared to the average requirement (AR), and as energy-adjusted intake (unit/7 MJ). AR is defined as ‘the lowest long-term intake level of a nutrient that will maintain a defined level of nutritional status in an individual’ (25).

### Basic characteristics

Information on age, years of education, body mass index (BMI), physical activity, smoking habits, and region of living was compared across adherence categories.

Age was divided into four age categories: aged 41–50, 51–60, 61–70, and 71–76 years. BMI was based on self-reported weight and height (kg/m^2^) (26) and was categorized as below normal weight (BMI <20), normal weight (BMI ≥20–24.9), overweight (BMI ≥25–29.9), and obese (BMI ≥30). Smoking habits were categorized as never, former, and current smokers. Physical activity was divided into low, medium, and high level based on a 10-point scale (27). Years of education was divided into three categories: <10 years of schooling, 10–12 years of schooling, or >12 years of schooling. Region of living in Norway was divided into six regions (Oslo, east, south, west, middle, and north).

### Statistical analysis

Median values with 25th and 75th percentiles or proportions (in percentages) were used to present the intake of food items and the basic characteristics of the participants. The food items (both those included and those not included in the Healthy Nordic Food Index) were analyzed using a nonparametric test for trend across ordered groups (nptrend in Stata), which is an extension to the Wilcoxon rank-sum test. Nptrend is testing for a linear trend over the three adherence categories, and it gives the two-sided *p*-value. It was applied to investigate if the daily intake of food items/nutrients, both as absolute measures and as energy-adjusted measures, was linearly associated with adherence categories (low, medium and high).

The same trend test, in addition to multinomial logistic regression models with the index category as the dependent variable and the low adherence category used as the reference category, was used to analyze associations between adherence categories, and basic demographic and lifestyle characteristics.

Multinomial logistic regression can be used when the outcome variable has more than two categories (28). We found it appropriate to treat the index score variable as categorical instead of ordered for the regression analysis to fit two models comparing medium adherence with low adherence and high adherence with low adherence. Since the outcome variable has three categories, the estimates from the multinomial logistic regression models are given as relative risk ratios (RRR) with 95% confidence intervals.

All regression models were adjusted for energy intake, age, and subcohort. The subcohorts (*n* = 5) were defined in batches with similar FFQs and time of recruitment. As the data were collected over a period of almost 10 years, some questions have been removed or added, due to the introduction of new foods, discontinuation of foods, or new study hypotheses generated for the subcohorts. A mutually adjusted model that also included education, BMI, physical activity, smoking status, and region of living was applied. All analyses were conducted using the software Stata/MP version 14.0. The significance criterion was set to 5% (*p* < 0.05).

## Results

The number of food items from the FFQ that was included in the calculation of the six index food groups varied from 12 items in the fish category to 2 items in the root vegetables and the cabbage categories, and a single item in the apples/pears, whole grain bread and breakfast cereals categories (Table 1). There were 81,516 women included in the final analyses, distributed as follows across adherence categories: low adherence (score 0–1) 22.8%, medium adherence (score 2–3) 49.0%, and high adherence (score 4–6) 28.2% (Table 2). The intake of all food groups incorporated in the Healthy Nordic Food Index is presented in Table 2. By design, all incorporated food groups increased across adherence categories (*p* < 0.001 for all food groups), with the biggest difference in the food group apples/pears ranging from a median intake of 20 gram/day to 140 gram/day in low and high adherers, respectively. The increment from medium to high adherers was larger than from low to medium adherers for all food groups incorporated in the index.

**Table 2:** Consumption of foods (gram/day) in the Healthy Nordic Food Index in the low-, medium-, and high-adherence categories in the Norwegian Women and Cancer cohort

Healthy Nordic Food Index components (gram/day)	All women		Healthy Nordic Food Index score					
	
	*n* = 81,516		0–1 (22.8%)		2–3 (49.0%)		4–6 (28.2%)	
			
	Median	P25–P75**	Median	P25–P75	Median	P25–P75	Median	P25–P75
Fish*	48	29–74	29	17–41	47	29–71	69	52–96
Root* vegetables	40	21.1–74.6	17.9	9.3–30.7	38.4	23.1–67.1	69	51.9–97.8
Cabbage*	22	10–45	11	5–19	22	10–43	44	25–67
Apples/pears*	60	20–140	20	9–60	60	20–140	140	60-140
Whole grain bread*	100	100–180	100	34–100	100	100–180	180	100–180
Breakfast cereals*	0	0–21	0	0–0	0	0–21	21	0–31

* Corresponds to a significant (*p* < 0.001) nonparametric test for trend over ordered groups.

** 25th and 75th percentile.

Intake of energy and macronutrients is presented in Table 3. Participants in the high-adherence category had a higher intake of energy (8.1 MJ in subjects with high adherence, 6.8 MJ medium adherence, 5.8 MJ low adherence) (*p* < 0.001). Although statistically highly significantly related, E% from proteins was only weakly associated with adherence categories, whereas E% from carbohydrates increased slightly, and E% from total-, saturated-, polyunsaturated-, monounsaturated- and trans-fatty acids, and from alcohol slightly decreased across adherence categories (*p* < 0.001 for all relationships).

**Table 3:** Consumption of energy and macronutrients in the low-, medium- and high-adherence categories in the Norwegian Women and Cancer cohort

Energy and macronutrients	All women		Healthy Nordic Food Index score						*p*-value (direction of association)*
	
	*n* = 81 516		0–1		2–3		4–6		
			
	Median	P25–P75**	Median	P25–P75	Median	P25–P75	Median	P25–P75	
Energy (MJ)	7.0	5.8–8.2	5.8	4.9–6.9	6.8	5.8–8.0	8.1	7.0–9.3	<0.001 (+)
Protein (E%)	18.1	16.5–19.9	17.9	16.2–19.7	18.2	16.6–20.0	18.2	16.8–19.8	<0.001 (+)
Carbohydrates (E%)	46.2	42.4–50.0	45.1	41.1–49.0	46.1	42.3–49.9	47.2	43.6–50.0	<0.001 (+)
Total fat (E%)	33.3	30.0–36.7	34.5	31.0–38.0	33.4	30.1–36.7	32.4	29.2–35.2	<0.001 (-)
Saturated fat (E%)	13.2	11.6–14.8	13.9	12.2–15.6	13.2	11.7–14.8	12.6	11.2–14.2	<0.001(-)
Polyunsaturated fat (E%)	5.8	4.9–7.0	5.8	4.8–7.0	5.8	4.9–7.0	5.8	4.9–6.8	<0.005 (-)
Monounsaturated fat (E%)	10.4	9.2–11.7	10.8	9.6–12.2	10.4	9.2–11.6	10.1	9.2–11.7	<0.001 (-)
Trans fatty acids (E%)	0.6	0.5–0.7	0.7	0.5 –0.8	0.6	0.5–0.7	0.6	0.5–0.7	<0.001 (-)
Alcohol (E%)	0.8	0.2–2.2	1.0	0.3–2.7	0.8	0.2–2.2	0.7	0.2–1.8	<0.001 (-)

* *p*-value generated form a nonparametric test for trend over ordered groups, (+) relates to a positive trend over adherence categories, and (-) relates to an inverse trend over adherence categories.

** 25^th^ and 75^th^ percentile.

Comparison of absolute intake and energy-adjusted intake of food items/nutrients not included in the index is presented in Table 4. Absolute intake of fiber, micronutrients, sodium, red meat and processed meat, added sugar, fruits and vegetables, milk and milk products, chicken, and potatoes increased with index category (*p* < 0.001 for all food items and nutrients). The differences in intake became less pronounced after energy adjustment but were still profound for fruits and vegetables, whereas the association with red meat and processed meat and added sugar became inversely associated with a high index category. The difference between absolute intake and energy-adjusted intake of red meat and processed meat increased from a difference of absolute intake of 5 gram/day (from 89 to 94 gram/day) between low- and high-adherence categories to a difference of 27 gram/7 MJ (from 108 to 81 gram) between the low- and high-adherence categories after energy adjustment (*p* < 0.001). The percentage of total fruits and vegetables covered by the items included in the index (cabbages, root vegetables, and apples/pears) varied across the adherence categories from 39.9% coverage in the low-adherence category, 49.7% coverage in the medium-adherence category, to 51.8% in the high-adherence category (results not presented). Participant characteristics in the low-, medium-, and high-adherence categories are presented in Table 5. The high adherers tended to be older, be more educated, have higher BMI, be more physically active, and be non-smokers (*p* < 0.01 for trend over categories for all characteristics).

**Table 4:** Comparison of absolute and energy adjusted intake of foods/nutrients not included in the calculation of the Healthy Nordic Food Index in the low-, medium-, and high-adherence categories in the Norwegian Women and Cancer cohort

Energy, foods, and nutrients	Absolute intake (unit/day)									Energy adjusted (unit/7 MJ)								
	
	All women		Healthy Nordic Food Index category						*p*-value (direction of association)*	All women		Healthy Nordic Food Index category						*p*-value (direction of association)*
			
	*n* = 81 516		0–1		2–3		4–6			*n* = 81 516		0–1		2–3		4–6		
							
	Median	P25–P75*	Median	P25–P75*	Median	P25–P75*	Median	P25–P75*		Median	P25–P75*	Median	P25–P75*	Median	P25–P75*	Median	P25–P75*	
Fiber (g)	21	17–26	16	13–18	21	18–24	27	24–31	<0.001 (+)	21	18–24	18	16–21	21	19–24	23	21–26	<0.001 (+)
Zinc (mg)	9	7–10	7	6–9	9	7–10	10	9–12	<0.001 (+)	9	8–10	9	8–10	9	8–10	8	8–9	<0.001 (–)
Selenium (µg/d)	58	46–71	45	37–55	57	47–69	70	59–84	<0.001 (+)	58	49–69	54	46–64	58	49– 70	61	52–72	<0.001 (+)
Iron (mg)	9	7–11	7	6–9	9	7–10	11	9–12	<0.001 (+)	9	8–10	9	8–10	9	8–10	9	8–10	<0.001 (+)
Folate (µg/d)	178	145–218	140	115–169	175	147–208	218	186–258	<0.001 (+)	178	157–206	167	146–192	178	156– 205	189	167– 216	<0.001 (+)
Vitamin D (µg/d)	6	4–12	4	3–7	6	4–11	8	6–15	<0.001 (+)	6	4–11	5	4–8	6	4–11	7	5–13	<0.001 (+)
Sodium (mg)	2322	1912–2783	1950	1609–2310	2305	1927–2713	2692	2282–3147	<0.001 (+)	2346	2132–2571	2346	2123–2577	2354	2135–2585	2334	2134–2542	<0.001 (–)
Red meat and processed meat (g)	91	63–124	89	61–121	91	63–124	94	64–126	<0.001 (+)	92	66–122	108	78–141	93	67–122	81	58–106	<0.001 (–)
Added sugar (g)	20	13–31	18	11–28	20	13–30	23	16–33	<0.001 (+)	21	14–29	22	14–32	21	14–30	20	14–27	<0.001 (-)

**Table 5:** Participant characteristics in the low, medium and high Healthy Nordic Food Index adherence category in the Norwegian Women and Cancer cohort (percentage distribution)

Basic characteristics	All women		Healthy Nordic Food Index score		
	
	*n* = 81,516	0–1 points *n* = 18,510	2–3 points *n* = 40,038	4–6 points *n* = 22,968	*p*-value*
	
	%	%	%	%	
**Age**					<0.001
41–50	46.7	52.9	46.6	41.8	
51–60	44.4	40.1	44.0	48.6	
61–70	8.5	6.6	9.0	9.2	
71–76	0.4	0.4	0.4	0.5	
**Education**					<0.001
<10	23.7	24.8	24.5	21.5	
10–12	34.6	36.6	34.5	33.4	
>12	41.7	38.6	41.0	45.2	
**BMI (kg/m^2^)**					0.003
<20	6.5	7.2	6.4	6.1	
≥20–24.9	53.9	54.6	53.3	54.2	
≥25–29.9	30.3	28.7	30.8	30.7	
≥30	9.4	9.6	9.6	9.0	
**Physical activity**					<0.001
Low	12.8	17.7	12.9	8.9	
Moderate	72.7	70.9	73.3	73.0	
High	14.5	11.4	13.9	18.1	
**Smoking status**					<0.001
Never	37.1	33.6	36.7	40.4	
Former	33.6	30.7	33.6	35.9	
Current	29.3	35.7	29.6	23.7	
**Region of living**					
Oslo	9.2	11.7	8.9	7.8	
East	36.0	39.3	35.7	34.0	
South	4.8	4.7	4.8	4.9	
West	21.6	17.5	21.0	26.0	
Middle	7.9	8.7	7.8	7.3	
North	20.5	18.2	21.9	20.0	

Percentage distribution by columns.

* *p*-value from the nonparametric test for trend over ordered groups.

BMI, body mass index.

The relative risk ratios from the multinomial regression analysis are presented in Table 6. The mutually adjusted model showed a greater likelihood of being in the high-adherence category if reporting a higher age and having more than 12 years of schooling (RRR 1.50, 95% CI 1.41–1.59). Being overweight (BMI ≥25–29.9) relative to being in the normal BMI category (≥20–24.9) increased the likelihood of being a high adherer with 32% (RRR 1.32, 95% CI 1.26–1.39). High level of physical activity increased the likelihood of being a high adherer by about 2.63 times (95% CI 2.41–2.87), and being a current smoker gave a 33% reduced likelihood of being in the high-adherence category relative to never having smoked (RRR 0.67, 95 % CI 0.63–0.71). Relatively to women who live in the Norwegian capital Oslo, women living in the western part (RRR 1.91, 95 % CI 1.76–2.09) or in the northern parts (RRR 1.76, 95 % CI 1.60–1.92) were more likely to be high adherers.

**Table 6:** Relative risk ratios for medium and high Healthy Nordic Food Index adherence category (with low adherence category as reference) according to non-dietary factors in the Norwegian Women and Cancer cohort

	Medium adherence				High adherence			
	
	Energy adjusted		Mutually adjusted		Energy adjusted		Mutually adjusted	
			
	RRR*	95% CI	RRR	95% CI	RRR	95% CI	RRR	95% CI
**Age**								
41–50	1				1			
51–60	1.42	1.37–1.48	1.42	1.36–1.47	2.00	1.91–2.10	2.03	1.94–2.13
61–70	1.91	1.78–2.10	1.87	1.74–2.02	2.84	2.61–3.10	2.89	2.65–3.16
71–76	1.83	1.36–2.46	1.93	1.43–2.60	3.12	2.22–4.40	3.48	2.46–4.92
**Education**								
<10	1				1			
10–12	0.91	0.87–0.96	1.03	0.98–1.08	0.98	0.92–1.04	1.18	1.11– 1.26
>12	0.99	0.94–1.04	1.16	1.10–1.22	1.17	1.11–1.24	1.50	1.41–1.59
**BMI (kg/m^2^)**								
<20	0.80	0.75–0.87	0.85	0.78–0.91	0.63	0.57–0.69	0.70	0.64–0.77
≥20–24.9	1		1		1		1	
≥25–29.9	1.22	1.17–1.27	1.19	1.14–1.24	1.35	1.29–1.42	1.32	1.26–1.39
≥30	1.16	1.09–1.24	1.18	1.11–1.26	1.22	1.13–1.31	1.30	1.20–1.41
**Physical activity**								
Low	1				1			
Moderate	1.29	1.22–1.35	1.34	1.28–1.41	1.73	1.62–1.85	1.83	1.71–1.97
High	1.48	1.38–1.59	1.59	1.48–1.70	2.35	2.16–2.56	2.63	2.41–2.87
**Smoking status**								
Never	1				1			
Former	1.08	1.04–1.13	1.10	1.05–1.15	1.13	1.07–1.19	1.18	1.12–1.25
Current	0.79	0.75–0.82	0.87	0.83–0.91	0.55	0.52–0.58	0.67	0.63–0.71
**Region of living**								
Oslo	1				1			
East	1.13	1.06–1.20	1.14	1.07–1.22	1.18	1.09–1.27	1.20	1.11–1.30
South	1.19	1.08–1.32	1.20	1.08–1.32	1.27	1.13–1.44	1.29	1.14–1.46
West	1.42	1.32–1.52	1.45	1.35–1.55	1.82	1.67–1.98	1.91	1.76–2.09
Middle	1.12	1.03–1.21	1.13	1.04–1.23	1.14	1.03–1.26	1.18	1.06–1.31
North	1.61	1.50–1.73	1.58	1.47–1.70	1.77	1.62–1.93	1.76	1.60–1.92

* Relative risk ratios from multinomial logistic regression.

RRR, relative risk ratios; BMI, body mass index.

## Discussion

The Healthy Nordic Food Index was adapted to the data in the NOWAC cohort. Absolute consumption of the index food groups in the NOWAC cohort seems to be higher than for similar food groups in the Swedish Women’s Lifestyle and Health cohort, and to the women in The Diet, Cancer and Health study (6, 7). Whether this reflects an actual difference in intake between countries, or is due to different assessment or criterion in the quantification of food intake in the FFQs, has not been investigated. However, compared to consumer surveys on household level and national 24-h dietary recall surveys in Norway, the intake of the index food groups reported in the NOWAC cohort seems reasonable (29). The macronutrient distribution was quite similar across adherence categories and within the Nordic Nutrition Recommendations (6, 7, 25). This is similar to what has been found in other studies on the Healthy Nordic Food Index (6, 7). Compared to low and medium adherence, high adherence coincided with a higher energy intake, a higher absolute intake of both healthy and less healthy foods, and a higher intake of foods with no clear association with beneficial health outcomes. Median intake in all adherence categories was within the Nordic Nutrition Recommendations for alcohol, carbohydrates, proteins, total fat, monounsaturated fat and polyunsaturated fat, but the consumption of saturated fat was higher than recommended in all adherence categories (25). As the high-adherence category had a higher absolute intake of some micronutrients, they were more likely to meet the average requirements for vitamins and minerals (25). The average requirement for zinc and selenium was met by all adherence categories, but only the high-adherence category met the average requirement for iron, folate, and vitamin D. Participants in the high-adherence category exceeded the upper limit for sodium. These results confirm and extend findings in previous studies, which link a high index score with higher food intake in general, and with a higher intake of both healthy foods and foods considered less healthy (6, 7). However, after energy adjustment, high adherers still had higher intake of fiber, micronutrients (except zinc), and fruits and vegetables, but zinc and the food items/nutrients considered less healthy (i.e. red meat and processed meat, added sugar, and sodium) and the foods with no clear health effect were inversely associated with a high index score.

Even though there were highly significant associations for all foods and nutrients analyzed, some are not considered to be of any clinical importance. The marginal differences in actual intake between adherence categories for these food items were statistically significantly associated only because of the high number of participants in the study. Nevertheless, it shows that the index does not merely measure a higher intake of all foods, but that high adherence is associated with better dietary quality. The association between high adherence to a healthy Nordic diet and higher intake of healthy foods, but not with a higher intake of meat and sweets, is supported by Bjørnarå et al. in a Norwegian study on the New Nordic Diet (5). Furthermore, the higher fraction of the healthy Nordic fruits and vegetables in the diet among medium and high adherers compared to low adherers shows that the index measures a healthy Nordic diet and not only a healthy diet. However, it also shows that low adherers of the index get a higher fraction of their total fruits and vegetables from food items outside the index. As these food items, such as tomatoes, oranges, and salad, also have anticipated health benefits, it should be taken into consideration in future studies on the association between the index and health outcomes.

High adherers were more physically active, had higher education, were older and were less likely current smokers. This is in accordance with what was found in the previous studies on the Healthy Nordic Food Index, as well as in studies on the Baltic Sea Diet Score and in relation to the New Nordic Diet (6–9). The association with BMI and adherence category was positive even though the low-adherence category had the highest proportion of women in both the lowest and highest BMI categories. A positive association between BMI and adherence category was found in the Swedish Women Lifestyle and Health study, whereas a high adherence score was related to lower BMI in the New Nordic Diet, as it is in relation the Mediterranean diet (6, 9, 30). In the NOWAC cohort, BMI has been identified as a predominant factor in explaining weight loss attempts, and women trying to lose weight reported a diet with less fat and more fiber, fruits, and vegetables compared to women not trying to lose weight (31). This may explain why we find that high BMI is associated with high adherence.

Women living in the west and north had a higher likelihood of being in the high-adherence category than women living in Oslo. These were the regions with the highest intake of fish, in particular the northern region. The high fish consumption in the northern parts of Norway has been confirmed in national dietary surveys (32). West and north also had a higher intake of root vegetables, possibly reflecting a more traditional dietary pattern in these regions, as the total intake of fruits and vegetables were higher in Oslo compared to north (median 322 gram/day vs. 259 gram/day) and about the same as in the west (330 gram/day). The type of fruits and vegetables more commonly consumed in Oslo might be of a more exotic kind as it is the capital and assumedly more influenced by trends and immigration. The assortment of imported fruits and vegetables is therefore probably better in Oslo than in the rest of the country.

### Strengths and limitations

The construction of the index is based on the median of the index variables, as in previous studies on the Healthy Nordic Food Index and the Mediterranean Diet Score (6, 7). Other indices use other scoring criteria such as quintiles or recommended values (4). One could argue that the use of the median criteria will simplify the information to a greater extent compared to other methods. However, The Dietary Patterns Methods Project (4) has made standardized methods for several indices using different scoring criteria with the aim of comparing their ability to capture a healthy diet and their association with mortality. They found that all indices captured the essence of a healthy diet, and the associations with reduced mortality were of similar strength. Hence, they did not recommend one dietary pattern over the other, and neither any particular scoring method in the construction of an index. In addition, when considering the positive health effects associated with the Healthy Nordic Food Index, it seems that the use of the median criteria is an acceptable method. The median cutoff is quite robust against misclassification of extreme values and might be appropriate when considering the accuracy of the FFQ data.

The use of FFQ is likely to introduce errors. These could be both random and systematic. As this FFQ has more questions concerning fish intake compared to the other index food groups, this might introduce overreporting of fish. We have to assume that overreporting due to a higher number of FFQ questions will affect all respondents to the same degree. This will result in a higher intake, but also a higher median cutoff value, and hence not influence the ranking to a major degree. Overreporting of fiber intake has been found in a NOWAC validation study, and overreporting of healthy foods is a well-known challenge with FFQs. If overreporting of healthy foods is systematically related to factors associated with the adherence categories (i.e. education and physical activity), it could bias the association between adherence category and other factors. It is a limitation that the FFQ was not initially designed to assess compliance with a healthy Nordic diet and thus does not capture all relevant food groups such as wild berries (i.e. cloudberries, blueberries, and raspberries), rye and oatmeal-specific whole grain, game, and rapeseed oil (18). However, the intake of foods such as wild berries and game in the general population was not high (29), neither was the intake of rapeseed oil at the time of data collection (33). Even though these are relevant foods in line with the rationale of the index and are relevant in promotion of a healthy Nordic diet, it is not likely that questions about these food items in the FFQ could have enhanced the precision or validity of the index as a measurement tool for a healthy Nordic diet, as most women would not have had a measurable intake. In relation to the Healthy Nordic Food Index, it seems that the six incorporated food items are sufficient to find associations with health outcomes and therefore is a valid tool. It cannot, however, be ruled out that the associations could have been even stronger and more consistent with the inclusion of more healthy Nordic foods. The index food groups ‘whole grain bread’ and ‘breakfast cereals’ are based on single question from the FFQ. It is likely that a more detailed assessment of types of whole grain bread and whole grain products in the breakfast cereals category would give a more precise assessment of the type and amount of whole grain in the diet. However, in a study part of the NOWAC cohort, it was found that whole grain bread captured 84% of the total whole grain consumption in Norwegian women, and approximately 80% of the grains in the cereal category were whole grains (34). Differences in the food components included in the index could affect the associated health outcomes in unknown directions and thereby the comparability of the index between countries. This might be particularly relevant for the index food items that include whole grains as there are some cultural differences between the types of grains commonly consumed in the Scandinavian countries. Danish women consume mostly rye, whereas wheat is the most commonly consumed grain in Norway (34).

The FFQ has been validated through several studies (21, 24, 35, 37). Measurement of serum phospholipids showed that fatty fish intake was reflected in serum (36). A repeated 24-h dietary recalls study (24) found that the FFQ gave a good ranking (Spearman’s correlation coefficient) of the participants’ intake of foods/drinks consumed frequently (e.g. coffee and milk) and fairly good for macronutrients, but weaker for foods infrequently eaten (e.g. desserts) and for some micronutrients. The FFQ performed well on ranking high and low consumers when compared to recall data, and for the purpose of this study, an adequate ranking of participants is more important than estimating the absolute intake. The food groups in the validation study are not completely overlapping the food items incorporated in the index except for fish, which had a Spearman’s correlation coefficient of 0.26 (24). The study also showed an underestimation of energy, fat, added sugar, and alcohol in the FFQ when compared to the 24-h dietary recalls, whereas fiber intake was overestimated compared to the 24-h dietary recalls. A test–retest study on the reproducibility of the FFQ concluded that the FFQ performed within the range described for comparable instruments (21). The large sample size also gives strength to the study as it is representative of the women in Norway at the time of data collection (35).

An advantage of using the *a priori* approach (which is hypothesis-driven based on assumptions of the foods that are included) is that the index is analytically simple to construct, and the results can more easily be compared to other studies than, for instance, data-driven explorative constructs (4). The benefit with measuring dietary patterns and dietary quality is that it adds the possibility of capturing health effects that might not be detectable for the single food component alone, due to the synergistic and combined effects of the components of the included food items (38). In addition, a dietary pattern is more comparable to what people eat, as we do not live eating single food item.

## Conclusion

This study links high adherence to a healthy Nordic diet, measured by the Healthy Nordic Food Index to a higher food and energy intake, and to a higher intake of some essential micronutrients. Trend analysis showed a positive relationship between both healthy and less healthy foods and higher adherence categories, but energy adjustment of potential confounding foods removed associations between high adherence and less healthy foods. The results point to an overall better composition of the diet among high adherers compared to low and medium adherers of the Healthy Nordic Food Index. However, both the absolute intake and the relative intake of Nordic and other fruits and vegetables suggest that the index captures Nordic foods and not just healthy foods and lifestyle in general. Furthermore, the healthy Nordic foods accounted for a larger fraction of the diet among high adherers, at the expense of other healthy food items (i.e. salad, tomatoes, oranges, and other vegetables). High adherence was associated with a healthier lifestyle, a higher level of education, and older age. This clustering of healthy lifestyle factors and a better dietary composition among high adherers should be taken into account in further studies on the Healthy Nordic Food Index and health outcomes.
