# Corrigendum

**DOI:** 10.29219/fnr.v68.10708

**Published:** 2024-05-08

**Authors:** Torill M. Enget Jensen, Tonje Braaten, Bjarne Koster Jacobsen, Runa Borgund Barnung, Anja Olsen, Guri Skeie

**Affiliations:** 1 Department of Community Medicine, Faculty of Health Sciences, UiT-The Arctic University of Norway, Tromsø, Norway; 2 Danish Cancer Society Research Center, Institute of Cancer Epidemiology, Danish Cancer Society, Copenhagen, Denmark

In the published article, the final portion of Table 4 was not published, specifically the last four lines of Table 4.

**Table 4:** Comparison of absolute and energy-adjusted intake of foods/nutrients not included in the calculation of the Healthy Nordic Food Index in the low, medium, and high adherence categories in the Norwegian Women and Cancer cohort

Energy, foods and nutrients	Absolute intake (unit/day)									Energy-adjusted (unit/7MJ)								
	All women *n* = 81,516		Healthy Nordic Food Index category						P (direction of association)*	All women *n* = 81,516		Healthy Nordic Food Index category						P (direction of association)*
			0–1		2–3		4–6					0–1		2–3		4–6		
	Median	P25–P75	Median	P25–P75	Median	P25–P75	Median	P25–P75		Median	P25–P75	Median	P25–P75	Median	P25–P75	Median	P25–P75	
Fibre (g)	21	17–26	16	13–18	21	18–24	27	24–31	<0.001 (+)	21	18–24	18	16–21	21	19–24	23	21–26	<0.001 (+)
Zinc (mg)	9	7–10	7	6–9	9	7–10	10	9–12	<0.001 (+)	9	8–10	9	8–10	9	8–10	8	8–9	<0.001 (–)
Selenium (µg/day)	58	46–71	45	37–55	57	47–69	70	59–84	<0.001 (+)	58	49–69	54	46–64	58	49–70	61	52–72	<0.001 (+)
Iron (mg)	9	7–11	7	6–9	9	7–10	11	9–12	<0.001 (+)	9	8–10	9	8–10	9	8–10	9	8–10	<0.001 (+)
Folate (µg/day)	178	145–218	140	115–169	175	147–208	218	186–258	<0.001 (+)	178	157–206	167	146–192	178	156–205	189	167–216	<0.001 (+)
Vitamin D (µg/day)	6	4–12	4	3–7	6	4–11	8	6–15	<0.001 (+)	6	4–11	5	4–8	6	4–11	7	5–13	<0.001 (+)
Sodium (mg)	2,322	1,912–2,783	1,950	1,609–2,310	2,305	1,927–2,713	2,692	2,282–3,147	<0.001 (+)	2,346	2,132–2,571	2,346	2,123–2,577	2,354	2,135–2,585	2,334	2,134–2,542	<0.001 (–)
Red-and processed meat (g)	91	63–124	89	61–121	91	63–124	94	64–126	<0.001 (+)	92	66–122	108	78–141	93	67–122	81	58–106	<0.001 (–)
Added sugar (g)	20	13–31	18	11–28	20	13–30	23	16–33	<0.001 (+)	21	14–29	22	14–32	21	14–30	20	14–27	<0.001 (–)
Fruit/vegetables (g)	305	203–444	183	115–258	296	213–403	456	344–593	<0.001 (+)	309	210–440	217	138–315	303	213–429	397	294–528	<0.001 (+)
Milk and milk products (g)	219	108–360	185	89–308	217	107–352	245	129–438	<0.001 (+)	218	115–374	224	110–389	220	115–373	213	118–360	<0.001 (–)
Chicken (g)	16	6–16	6	6–16	16	6–16	16	6–16	<0.001 (+)	13	6–20	13	6–22	13	6–20	12	5–19	<0.001 (–)
Potatoes (g)	126	50–126	63	50–126	126	50–126	126	50–126	<0.001 (+)	100	51–143	96	49–158	105	51–143	96	50–143	<0.001 (–)

* *p*-value generated from a nonparametric test for trend over ordered groups, (+) relates to a positive trend over adherence categories, and (–) relates to an inverse trend over adherence categories.

Additionally, there was an inaccuracy in the description of the scoring methodology used to calculate the Healthy Nordic Food Index. It was incorrectly stated that an intake below the median is scored as zero points, and an intake at or above the median is scored as one point. The scoring was conducted such that an intake at or below the median received a score of zero, and an intake above the median was awarded a score of 1. This has now been updated (page 4 in the publication) as follows: ‘To compute the index score for each participant, the intake of each food item included in the index was divided by the cohort median to assign each participant either a score of 1 if they were above the study median, or a score of 0 if equal to or below the study median’.

The authors sincerely regret any inconvenience this may have caused.

These discrepancies do not affect any of the numerical data or the results presented in the tables. The overall results and conclusions are upheld.

